# MicroRNA-6744-5p promotes anoikis in breast cancer and directly targets NAT1 enzyme

**DOI:** 10.20892/j.issn.2095-3941.2019.0010

**Published:** 2020-02-15

**Authors:** Sharan Malagobadan, Chai San Ho, Noor Hasima Nagoor

**Affiliations:** ^1^Institute of Biological Sciences (Genetics & Molecular Biology), Faculty of Science, University of Malaya, Kuala Lumpur 50603, Malaysia; ^2^Center for Research in Biotechnology for Agriculture (CEBAR), University of Malaya, Kuala Lumpur 50603, Malaysia

**Keywords:** Anoikis, miRNA, breast cancer, metastasis, NAT1

## Abstract

**Objective:** Anoikis is apoptosis that is induced when cells detach from the extracellular matrix and neighboring cells. As anoikis serves as a regulatory barrier, cancer cells often acquire resistance towards anoikis during tumorigenesis to become metastatic. MicroRNAs (miRNAs) are short strand RNA molecules that regulate genes post-transcriptionally by binding to mRNAs and reducing the expression of its target genes. This study aimed to elucidate the role of a novel miRNA, miR-6744-5p, in regulating anoikis in breast cancer and identify its target gene.

**Methods:** An anoikis resistant variant of the luminal A type breast cancer MCF-7 cell line (MCF-7-AR) was generated by selecting and amplifying surviving cells after repeated exposure to growth in suspension. MiRNA microarray analysis identified a list of dysregulated miRNAs from which miR-6744-5p was chosen for overexpression and knockdown studies in MCF-7. Additionally, the miRNA was also overexpressed in a triple-negative breast cancer cell line, MDA-MB-231, to evaluate its ability to impair the metastatic potential of breast cancer cells.

**Results:** This study showed that overexpression and knockdown of miR-6744-5p in MCF-7 increased and decreased anoikis sensitivity, respectively. Similarly, overexpression of miR-6744-5p in MDA-MB-231 increased anoikis and also decreased tumor cell invasion *in vitro* and *in vivo*. Furthermore, NAT1 enzyme was identified and validated as the direct target of miR-6744-5p.

**Conclusions:** This study has proven the ability of miR-6744-5p to increase anoikis sensitivity in both luminal A and triple negative breast cancer cell lines, highlighting its therapeutic potential in treating breast cancer.

## Introduction

Breast cancer represents a major challenge in the field of cancer therapeutics, being the second most common cancer worldwide and the most common cancer affecting women^1^. Decisions regarding treatment options are often determined based on breast cancer types and the expression of breast cancer markers. As such, breast cancer can be categorized into three classifications, which are the luminal type (luminal A and luminal B), HER2-overexpressing type and triple-negative type^2^. However, these classifications continue to be updated and renewed in the face of new findings to include other cancer-related markers.

Anoikis is apoptosis that is induced when cells lose their attachment to the extracellular matrix (ECM) and surrounding cells^3^. The ECM not only acts as a structural scaffold to enable cellular attachment and localization but also presents various ligands to provide the necessary signals for cell surface receptors such as E-cadherin and integrins^4^. As this mechanism is mostly intact in the early stages of tumorigenesis, anoikis forms a regulatory barrier by preventing cancer cells from surviving the detachment from their primary location. However, cancer cells often acquire resistance to anoikis and undergo various changes such as epithelial-to-mesenchymal transition (EMT) to become more aggressive and metastatic.

MicroRNAs (miRNAs) are among the various non-coding RNAs that have been found to play critical roles in gene regulation. miRNAs are short strands of RNA, and they play an important function in post-transcriptional gene regulation by binding to the 3' UTR of target mRNA containing complementary sequences, causing either its degradation or translational impairment^5^. As miRNAs are necessary for regular cell functions, dysregulation of miRNA expression has been well documented in various abnormal conditions, especially cancer. As such, it is not surprising that miRNAs play direct roles in regulating various cancer cell phenotypes, particularly those relating to apoptosis and metastasis^6^. In terms of anoikis, studies have shown that numerous miRNAs promote or impede anoikis in cancer cells through the downregulation of their target genes^7^.

This study aimed to investigate how a novel miRNA regulates anoikis in breast cancer cells and determine its target protein. We have identified miR-6744-5p as a downregulated miRNA in an anoikis resistant sub-cell line (MCF-7-AR6) generated from MCF-7 breast cancer cells. *In vitro* studies showed that miR-6744-5p promotes anoikis when overexpressed in MCF-7 and triple-negative type MDA-MB-231 cells. Furthermore, we also demonstrated that overexpression of miR-6744-5p inhibits the invasiveness of MDA-MB-231, both *in vitro* and *in vivo*, using Transwell-invasion assays and a zebrafish metastasis model, respectively. Additionally, we have validated a xenobiotic metabolizing enzyme, N-acetyltransferase 1 (NAT1), as the direct target of miR-6744-5p. Overall, we have shown that overexpression of miR-6744-5p increases anoikis and reduces migration and invasion in breast cancer cell lines.

## Materials and methods

### Cell culture and maintenance

Two breast cancer cell lines, MCF-7 and MDA MB-231, were obtained from the American Type Culture Collection (Manassas, VA, USA). MCF-7 was cultured in α-MEM (Nacalai Tesque, Kyoto, Japan), while MDA-MB-231 was cultured in RPMI (Hyclone, Logan, UT, USA). Both cell culture media were supplemented with 10% fetal bovine serum (FBS) (Hyclone, UT, USA) and 1% penicillin/streptomycin mix (Lonza, Basel, Switzerland) unless otherwise stated. The cells were maintained in a carbon dioxide (CO_2_) incubator, with humidity set at 95%, temperature at 37 °C, and CO_2_ at 5%.

### Ethics approval

Experiments involving zebrafish were approved by the Faculty of Medicine Institutional Animal Care and Use Committee (IACUC), University of Malaya (Approval No. 2017-181108/IBS/R/SM).

### Generation of an anoikis resistant sub-cell line

MCF-7 cells were cultured in Costar® Ultra Low Attachment (ULA) plates (Corning, NY, USA) for 72 h to induce anoikis. The cells were then transferred to normal tissue culture plates to enable viable cells to attach and proliferate. After 24 h, the media was replaced to discard floating dead cells. After 6 cycles, the cells were transferred from suspension condition in the ULA plate to adherent condition in tissue culture plate to establish and propagate the MCF-7-AR6. The cells that did not attach and remained in suspension in tissue culture plate were confirmed to be non-viable using Trypan blue staining and removed from the plate by rinsing with phosphate-buffered saline (PBS). The cells were propagated to make stock and immediately used for the subsequent experiments, such as miRNA microarray, anoikis assay, caspase-3/7 assay, and wound healing assay within several passages. Whenever the stock had to be revived, anoikis- and caspase-3/7 assays were carried out to confirm the anoikis-resistance function of MCF-7-AR6 compared to MCF-7 before use in any experiments.

### Anoikis assay

Viable cells were measured using the CellTiter-Glo® Luminescent Cell Viability Assay kit (Promega, Madison, WI, USA). A total of 0.3 × 10^6^ cells of MCF-7 or MCF-7-AR6 were suspended in 1 mL of recommended media and transferred to a well in 24-well ULA plate for 48 h at 37 °C to induce anoikis. For transfected cells, at 48 h post-transfection, cells from each well of a 6-well tissue culture plate were trypsinized and mixed with spent media from respective wells. The cells were then centrifuged and resuspended in 2 mL of respective growth medium before being transferred into a well of a 6-well ULA plate for 48 h of incubation at 37 °C in suspension. Cell viability was measured by recording the luminescence using GloMax®-Multi Jr Single Tube Multimode Reader (Promega) according to the manufacturer’s protocol.

### Caspase-3/7 activity assay

Caspase-3/7 activity was detected using a Caspase-Glo® 3/7 Assay kit (Promega). A total of 0.3 × 10^6^ cells of MCF-7 or MCF-7-AR6 were suspended in 1 mL of recommended media and transferred to a well of a 24-well ULA plate for 24 h at 37 °C in suspension. For transfected cells, at 48 h post-transfection, cells from each well of a 6-well tissue culture plate were trypsinized and combined with spent media. The cells were then centrifuged and resuspended in 2 mL of respective growth medium before being transferred into a well of a 6-well ULA plate for 24 h at 37 °C to induce anoikis. The caspase-3/7 activity in each sample was then measured according to the manufacturer’s protocol after 1 h incubation in the dark at 25 ˚C using GloMax®-Multi Jr Single Tube Multimode Reader (Promega).

### Wound healing assay

Cells were grown to confluency in a 6-well tissue culture plate. A scratch was made on the monolayer by dragging a p200 pipette tip across the well. The well was rinsed twice with PBS to remove the dislodged cell debris and culture media supplemented with 1% FBS was added to the well. The wound gap was photographed at 40× magnification at 0 h and 24 h at a specific predefined point in the well. The relative wound area was measured using TScratch v1.0 software (ETH, Zurich, Switzerland).

### Total RNA extraction

Total RNA was extracted from cell pellets using a Qiagen miRNeasy mini kit (Qiagen, Hilden, Germany) based on the manufacturer’s protocol. Quality and quantity analysis of the extracted RNA was performed using a NanoDrop Spectrophotometer (Thermo Fisher Scientific, Waltham, MA, USA) for reverse transcription–quantitative polymerase chain reaction (RT-qPCR) and an Agilent 2200 TapeStation System (Agilent Technologies, Santa Clara, CA, USA) for miRNA microarray.

### miRNA microarray

To screen for miRNAs regulating anoikis resistance, the differential expression of miRNA for parental and anoikis resistant cell lines was analyzed using GeneChip® miRNA Arrays (Affymetrix, Santa Clara, CA, USA) based on the manufacturer’s recommended protocol. Affymetrix Transcriptome Analysis Console v3.0 (Affymetrix) was used to analyze the results using one-way between-subject analysis of variance (ANOVA), with an ANOVA *P*-value < 0.05, and fold change threshold set at >2.5 or <−2.5.

### RT-qPCR

Total RNA (1000 ng) was reverse transcribed and used to perform RT-qPCR for miR-6744-5p and small nuclear RNA (RNU6B) using TaqMan MicroRNA Assay (Applied Biosystems, Carlsbad, CA, USA) using the manufacturer’s recommended protocol. The fold change for each miRNA was calculated using the 2^−ΔΔCt^ method with RNU6B as the internal control. CFX96™ Real-Time PCR Detection System (Bio-Rad Laboratories, Hercules, CA, USA) and Bio-Rad CFX Manager™ v1.6 (Bio-Rad Laboratories) were used for the RT-qPCR and analysis.

### Transfection

Transfection was carried out 24 h after plating using Dharmafect 1 transfection reagent (GE Healthcare Dharmacon, Lafayette, CO, USA). miRIDIAN hsa-miR-6744-5p mimic and miRIDIAN miRNA Mimic Negative Control (GE Healthcare Dharmacon) were used for overexpression, and miRIDIAN hsa-miR-6744-5p hairpin inhibitor and miRIDIAN microRNA Hairpin Inhibitor Negative Control (GE Healthcare Dharmacon) were used for knockdown studies according to the manufacturer’s recommended protocol.

### Transwell-invasion assay

Transfected MDA-MB-231 cells were serum starved for 24 h before being trypsinized and counted. Each 24-well Transwell insert (8-µm pore size; BD Biosciences, Franklin Lakes, NJ, USA) was coated with 30 µg of Matrigel (Corning). Once the Matrigel had solidified, 0.2 × 10^6^ cells in 500 µL serum-free media were seeded into the upper chamber of the insert, while 1 mL of media supplemented with 20% FBS was added to the lower chamber. After 24 h of incubation at 37 °C, the non-invading cells were removed by rinsing the inserts in PBS and wiping the inside of the inserts with a cotton swab. To visualize the invading cells, the cells were fixed in 100% ethanol (Fisher Scientific) for 2 min and stained with methylene blue (Sigma Aldrich, St. Louis, MO, USA) solution [1% w/v in distilled water (dH_2_O)] for 20 min. After staining, 5 random fields in each insert were photographed at 100× magnification for every replicate, and the number of cells was counted using ImageJ v1.50i software [National Institutes of Health (NIH), Bethesda, MD, USA].

### Zebrafish metastasis assay

Transfected MDA-MB-231 cells were stained with 1,1'-dioctadecyl-3,3,3',3'-tetramethylindocarbo-cyanine perchlorate (DiI) stain (Invitrogen, Carlsbad, CA, USA) prior to microinjection. The cells were injected into the yolk of 48 h post-fertilization (hpf) zebrafish embryos using a FemtoJet Microinjector (Eppendorf, Hamburg, Germany) and an InjectMan NI 2 Micromanipulator (Eppendorf). The instruments were set to inject approximately 200 cells per injection into each embryo. Embryos were maintained in system water containing N-phenylthiourea (PTU) (Sigma Aldrich) in the dark for 48 h post-injection (hpi) at 33 °C. The embryos were anesthetized in benzocaine (Sigma Aldrich) and serial sections of the embryos were imaged using a Leica TCS SP5 II confocal microscope (Leica Microsystems, Wetzlar, Germany) to visualize the dissemination of DiI-stained MDA-MB-231 cells from the yolk into the body. The percentage of metastatic MDA-MB-231 cells (outside the yolk) was measured from the z-stack images using ImageJ v1.50i software.

### Western blot

Total protein extraction was carried out using RIPA buffer (Thermo Fisher Scientific) according to the manufacturer’s protocol 72 h after transfection. Protein samples were resolved in 12% sodium dodecyl sulfate polyacrylamide gel electrophoresis (SDS-PAGE) and transferred onto nitrocellulose membranes. Membranes were blocked and incubated with primary monoclonal antibodies against E-cadherin, glyceraldehyde 3-phosphate dehydrogenase (GAPDH) (Cell Signaling Technology (CST), Danvers, MA, USA) or NAT1 (Abcam, Cambridge, MA, USA) followed by secondary goat anti-rabbit IgG HRP-linked antibody (CST). Protein bands were visualized through chemiluminescence using WesternBright Quantum (Advansta Inc., San Jose, CA, USA), and band intensities were measured with ImageJ v1.50i software and normalized to GAPDH.

### *In silico* target prediction analysis

TargetscanHuman v7.1 online software was used to determine the predicted target of miR-6744-5p using default parameters. Predicted target site information was obtained from the analysis for dual-luciferase reporter assay.

### Dual-luciferase reporter assay

The 3'-UTR fragment of the wild-type *NAT1* gene containing the predicted targeting site of miR-6744-5p or a 3'-UTR fragment of the mutated *NAT1* gene with mutations within the predicted targeting site of miR-6744-5p were cloned into pmirGLO Dual-Luciferase miRNA Target Expression Vector (Promega). After 48 h of co-transfection with miR-6744 mimic or mimic negative control, normalized luciferase activity was measured according to the manufacturer’s protocol using the Dual-Luciferase® Reporter Assay System (Promega).

### Statistical analysis

Statistical differences were evaluated by paired Student’s *t*-tests and *P* < 0.05 was considered to be significant. Experiments were carried out with 3 independent replicates unless otherwise stated.

## Results

### MiR-6744-5p was downregulated in the anoikis resistant MCF-7-AR6 sub-cell line

**Figure 1:**
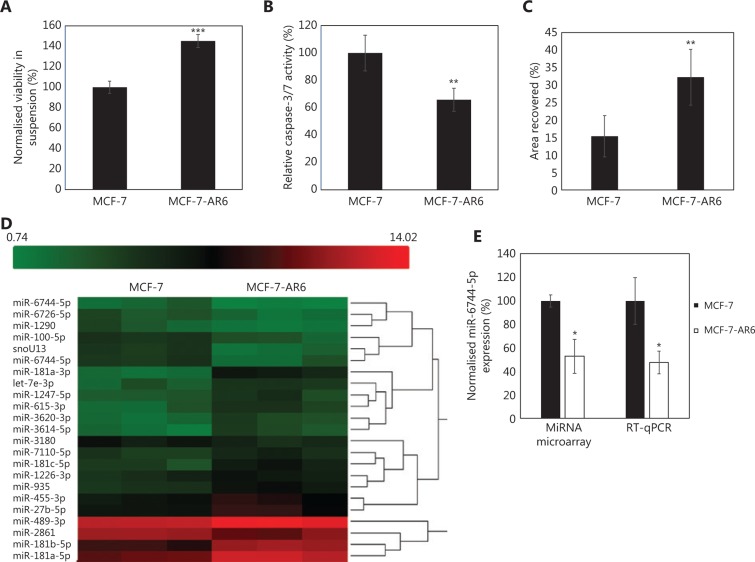
Downregulation of miR-6744-5p in anoikis resistant MCF-7-AR6. Comparison of MCF-7 and MCF-7-AR6 by measuring (A) viability after 48 h in suspension, (B) caspase-3/7 activity after 24 h in suspension and (C) scratch area recovery in wound healing assay after 24 h. (D) Hierarchical cluster heat map of miRNAs that are differentially expressed in MCF-7-AR6 when compared to the parental MCF-7. Red and green spectrum is used to represent signal strength, with red denoting high and green denoting low signal. The Affymetrix Transcriptome Analysis Console v3.0 was used to analyze the results using one-way between-subject analysis of variance (ANOVA), with ANOVA *P*-value < 0.05, and fold change >2.5 or <−2.5. (E) Downregulation of the selected miRNA, miR-6744-5p, was validated with reverse transcription-quantitative polymerase chain reaction (RT-qPCR). Data are presented as mean ± standard deviation (SD), and statistically significant differences compared to respective controls are denoted by (*) for *P* < 0.05, (**) for *P* < 0.01 or (***) for *P* < 0.001.

To elucidate miRNAs regulating anoikis in breast cancer, an anoikis resistant variant of the MCF-7 cell line, MCF-7-AR6 was generated. After 6 cycles, MCF-7-AR6 was only growth-competent in adherent conditions and was cultured on normal tissue culture plates. MCF-7-AR6 was compared to the parental MCF-7 cell line, and MCF-7-AR6 was found to be more resistant to anoikis. To determine anoikis resistance, anoikis was induced by subjecting the cells to suspension to measure cell viability and caspase-3/7 activity. Cell viability was measured in the surviving population after 48 h in suspension while caspase-3/7 activity was measured after 24 h in suspension. MCF-7-AR6 showed significantly higher viability (*P* < 0.001; **Figure 1A**) and lower caspase-3/7 activity (*P* = 0.006; **Figure 1B**) when compared to MCF-7. MCF-7-AR6 also showed increased migration compared to MCF-7 (*P* = 0.007; **Figure 1C**) in a wound healing assay. Next, miRNA microarray was performed to determine the changes in the miRNA expression in MCF-7-AR6 (**Figure 1D**). A total of 22 miRNAs were found to be differentially expressed with a fold change of >2.5 or <−2.5 (**Table 1**). From this list, an miRNA downregulated in MCF-7-AR6, miR-6744-5p, was chosen for further analysis owing to its possible novel role in regulating anoikis. RT-qPCR analysis provided quantitative confirmation for the downregulation of miR-6744-5p in MCF-7-AR6 (**Figure 1E**).

### Overexpression of miR-6744-5p in MCF-7 cell line increased anoikis sensitivity

**Figure 2:**
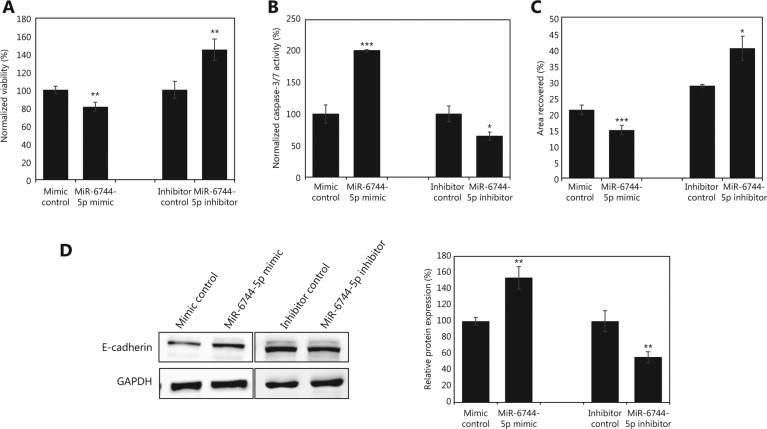
miR-6744-5p promotes anoikis in MCF-7. Effects of overexpression or knockdown of miR-6744-5p in MCF-7 measured by (A) viability assay after 48 h in suspension, (B) caspase-3/7 activity assay after 24 h in suspension and (C) wound healing assay after 24 h. (D) Western blot analysis of E-cadherin normalized to glyceraldehyde 3-phosphate dehydrogenase (GAPDH), and relative protein expression calculated from quantified band intensity. Data are presented as mean ± standard deviation (SD), and statistically significant differences compared to respective controls are denoted by (*) for *P* < 0.05, (**) for *P* < 0.01 or (***) for *P* < 0.001.

To understand the role of miR-6744-5p and determine why it was downregulated in MCF-7-AR6, overexpression and knockdown studies were performed in MCF-7 cells. Firstly, overexpression of miR-6744-5p reduced the viability of cells in suspension (*P* = 0.001; **Figure 2A**) and increased apoptosis when anoikis was induced (*P* < 0.001; **Figure 2B**). The opposite effects were observed following knockdown of miR-6744-5p. As expected, overexpression of miR-6744-5p also significantly reduced wound healing (*P* < 0.001) while knockdown increased wound area recovery (*P* = 0.028; **Figure 2C**). To further explore the possible role of miR-6744-5p in regulating anoikis, a Western blot was carried out to compare the expression of E-cadherin (**Figure 2D**), an important cell surface receptor mediating cell–cell interaction and contact inhibition. Expression of E-cadherin was increased when miR-6744-5p was overexpressed (*P* = 0.006) and decreased (*P* = 0.003) when miR-6744-5p was knocked-down.

### Overexpression of miR-6744-5p increased anoikis sensitivity and induced morphological changes in MDA-MB-231 cell line

**Figure 3:**
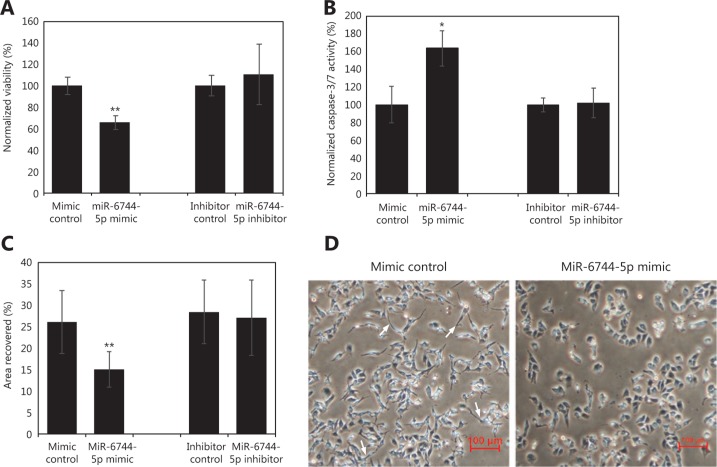
miR-6744-5p promotes anoikis in MDA-MB-231. Effects of overexpression of miR-6744-5p in MDA-MB-231 measured by (A) viability assay after 48 h in suspension, (B) caspase-3/7 activity assay after 24 h in suspension and (C) wound healing assay after 24 h. Data are presented as mean ± standard deviation (SD), and statistically significant differences compared to respective controls are denoted by (*) for *P* < 0.05 or (**) for *P* < 0.01. (D) Morphological changes of MDA-MB-231 cells observed 72 h post-transfection during overexpression of miR-6744-5p (white arrows indicate examples of spindle-like protruding edges).

To determine if miR-6744-5p played a functional role in other breast cancer types, a triple-negative cell line, MDA-MB-231, was chosen for overexpression and knockdown studies. MDA-MB-231 is an invasive breast cancer cell line that does not express E-cadherin. Similar to the findings from MCF-7, overexpression of miR-6744-5p decreased viability (**Figure 3A**) (*P* < 0.001), increased apoptosis (*P* = 0.017; **Figure 3B**) while in suspension, and inhibited wound closure (*P* = 0.009; **Figure 3C**). Additionally, overexpression also caused the cells to lose spindle-like protrusions on their edges and gain a relatively rounded structure (**Figure 3D**). Western blot showed E-cadherin was not expressed following the overexpression of miR-6744-5p (data were not shown).

### Overexpression of miR-6744-5p in MDA-MB-231 reduced invasiveness *in vitro* and *in vivo*

**Figure 4:**
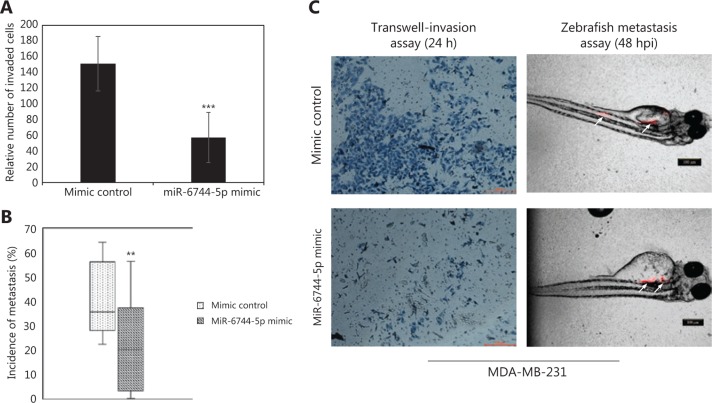
miR-6744-5p impedes invasiveness of MDA-MB-231. (A) The relative number of transfected MDA-MB-231 cells that invaded the Matrigel in a Transwell-invasion assay. (B) Incidence of metastasis in zebrafish model measured by percentage of DiI stained (red) MDA-MB-231 cells, transfected with mimic control (*n* = 10) or miR-6744-5p mimic (*n* = 12), that have metastasized out of the yolk to the rest of the body and tail. (C) Representative images of the Transwell-invasion assay after 24 h and zebrafish metastasis assay 48 h post-injection (hpi) (white arrows indicate the distribution of stained MDA-MB-231 cells). Data are presented as mean ± standard deviation (SD), and statistically significant differences compared to respective controls are denoted by (**) for *P* < 0.01 or (***) for *P* < 0.001.

As MDA-MB-231 is an invasive cell line, the impact of miR-6744-5p overexpression on invasiveness was investigated. Transwell-invasion assays showed that overexpression of miR-6744-5p significantly reduced invasion (*P* < 0.001; **Figure 4A**). In addition, an *in vivo* metastasis study using the zebrafish model^8^ was carried out, which showed that the overexpression of miR-6744-5p significantly reduced the incidence of metastasis (*P* = 0.009; **Figure 4B**), as demonstrated by the lower percentage of cells that have metastasized out of the zebrafish yolk injection site (**Figure 4C**).

### miR-6744-5p is validated to target NAT1 enzyme

**Figure 5:**
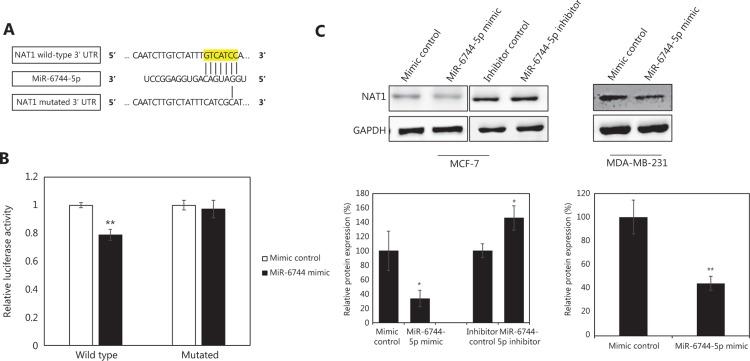
Validation of NAT1 targeting by miR-6744-5p. (A) The sequence of NAT1 wild-type 3' UTR with predicted targeting site containing base complementarity (highlighted in yellow) with miR-6744-5p and NAT1 mutated 3′ UTR. (B) Relative luciferase activity, normalized to *Renilla* luciferase activity, measured in cells transfected with wild type or mutated pmirGLO construct during overexpression or knockdown of miR-6744-5p. (C) Relative protein expression of NAT1 enzyme upon overexpression or knockdown in MCF-7 and knockdown in MDA-MB-231. Data are presented as mean ± standard deviation (SD), and statistically significant differences compared to respective controls are denoted by (*) for *P <* 0.05 or (**) for *P <* 0.01.

Using TargetscanHuman v7.1 software to determine putative target genes for miR-6744-5p, NAT1 enzyme was selected for further analysis due to the similarities between our *in vitro* findings and existing studies on NAT1 enzyme expression and breast cancer^9^. For validation of NAT1 enzyme targeting by miR-6744-5p, a fragment of 3' UTR of the *NAT1* gene containing either the wild-type sequence of the predicted miR-6744-5p target site or mutated sequence was cloned into the pmirGLO Dual-Luciferase miRNA Target Expression Vector (**Figure 5A**). Luciferase assay showed the overexpression of miR-6744-5p significantly reduced luciferase activity (*P* = 0.004) in cells co-transfected with the vector containing wild-type *NAT1* 3' UTR, whereas no significant difference was observed with the vector containing mutated *NAT1* 3' UTR (**Figure 5B**). Western blot provided additional confirmation for NAT1 targeting by miR-6744-5p, as NAT1 enzyme was found to be downregulated when miR-6744-5p was overexpressed in both MCF-7 (*P* = 0.024) and MDA-MB-231 (*P* = 0.003) cell lines (**Figure 5C**).

## Discussion

Resistance to anoikis in cancer cells is often accompanied by EMT and subsequently metastasis, making anoikis an early barrier to tumorigenesis. This study was initiated with MCF-7, a luminal A type breast cancer cell line, to generate a stable anoikis resistant sub-cell line (MCF-7-AR6). By comparing MCF-7-AR6 to MCF-7 using miRNA microarray, a list of miRNAs with possible roles in anoikis regulation was generated. Of these miRNAs with high fold change, many have been extensively studied in the context of anoikis or breast cancer, such as miR-181a-3p, miR-615-3p, miR-27b-5p, and miR-2861^10–13^. Notably, some of these miRNAs are known to play the paradoxical role of being oncogenic and tumor suppressive depending on the cell line. As such, miR-6744-5p was chosen for further analysis owing to its high fold change and novel role in breast cancer and anoikis. As miR-6744-5p was downregulated in MCF-7-AR6, it was hypothesized to play a tumor suppressive role by promoting anoikis. To test this hypothesis, miRNA overexpression and knockdown studies were carried out in MCF-7 and MDA-MB-231 cell lines.

Firstly, overexpression of miR-6744-5p in MCF-7 was found to promote anoikis, decrease migration, and increase the expression of E-cadherin, whereas knockdown achieved opposite outcomes. E-cadherin is an important epithelial marker and a cell surface receptor that regulates anoikis and cell–cell interaction. Its loss leads to EMT, which enables cancer cells to adopt mesenchymal-like characteristics. As the cells lose their epithelial characteristics, morphological changes can be observed, as cells show higher motility and increased anoikis resistance followed by metastasis^4^. As such, the increase in the expression of E-cadherin in MCF-7 upon overexpression of miR-6744-5p was in line with the predicted hypothesis as it prevents cells from surviving the disassociation from neighboring cells into an anchorage-independent condition.

Next, similar experiments were conducted using MDA-MB-231, a triple-negative breast cancer cell line lacking E-cadherin expression. The knockdown of miR-6744-5p did not result in any significant changes, which may be due to the inherently low expression of the miRNA in this cell line. On the other hand, overexpression of miR-6744-5p not only increased anoikis but also caused a change in cell morphology. The cells began to lose their spindle-like shape and acquire rounded edges. As overexpression of miR-6744-5p did not restore expression of E-cadherin in the MDA-MB-231 cell line, it can be suggested that the change in morphology was independent of E-cadherin.

Unlike MCF-7, MDA-MB-231 is a highly invasive breast cancer cell line^14^. As such, the effects of miR-6744-5p on invasiveness were tested using this cell line. It was found that miR-6744-5p significantly reduced invasiveness both *in vitro* and *in vivo*, shown in Transwell-invasion assays and a zebrafish metastasis model.

Finally, through *in silico* target prediction analysis and *in vitro* validations with luciferase assay and Western blot, it was confirmed that miR-6744-5p directly targets and downregulates NAT1, a xenobiotic-metabolizing enzyme. In breast cancer, high NAT1 expression has been correlated with the expression of estrogen receptor (ER) but without direct regulation by ER modulators^15,16^. Other studies have attempted knockdown of NAT1 enzyme in breast cancer cells, producing comparably identical *in vitro* results to that seen during the overexpression of miR-6744-5p in MDA-MB-231^9^. However, the link between the acetylation function of NAT1 enzyme and oncogenic phenotype observed when NAT1 is overexpressed is yet to be clarified^17^. Among the possible mechanisms by which NAT1 could contribute to anoikis resistance is by causing DNA damage through chemical carcinogenesis. As an enzyme that adds acetyl group to its substrate, NAT1 is involved in the conversion of 4-aminobiphenyl into DNA adduct, a carcinogenic compound that has been popularly associated with red meat consumption^18,19^. Moreover, a recent study has also unraveled evidence suggesting NAT1 inhibits reactive oxygen species (ROS) to suppress anoikis^20^.

It is important to note that miR-6744-5p is not the first miRNA discovered to target NAT1 enzyme in breast cancer. Endo and colleagues^21,22^ have published their findings and validation of miR-1290 targeting NAT1 enzyme using ER α-positive breast cancer tissue samples. Research on breast cancer and NAT1 enzyme has only begun to gain momentum, and it is expected that *NAT1*’s role as an oncogene will achieve further clarification in the near future.

Furthermore, the variable expression of miRNAs associated with breast cancer malignancies can also be utilized in early prediction and prognostic applications. For example, analysis of miRNAs regulating senescence and metastasis, miR-21, miR-23b, miR-190, and miR-200c, in plasma samples collected from breast cancer patients showed high correlation with cancer relapse and overall survival when compared to actual clinical data^23^. As such, the reduced expression of miR-6744-5p during the acquisition of anoikis resistance can be similarly monitored in breast cancer patients and it is potentially associated with chances of metastasis and overall survival.

## Conclusions

A novel miRNA that is downregulated during the acquisition of anoikis resistance in MCF-7 cell line has been identified. The overexpression of this miRNA, miR-6744-5p promotes anoikis in both luminal A and triple negative breast cancer cell lines, and miR-6744-5p directly target NAT1 enzyme, indicating its potential in breast cancer therapeutics and prognostics.

## Figures and Tables

**Table 1:** List of miRNAs found to be dysregulated in MCF-7-AR6 compared to MCF-7

MiRNA	Fold change	ANOVA *P*
MiR-6744-5p	−5.97	0.012
MiR-2861	−3.04	0.039
MiR-6744-3p	−2.96	0.017
miR-1290	−2.88	0.048
miR-6726-5p	−2.81	0.019
miR-100-5p	−2.67	0.012
miR-3180	−2.58	0.041
miR-455-3p	2.54	0.036
miR-1226-3p	2.54	0.045
miR-7110-5p	2.54	0.045
miR-3620-3p	2.8	0.012
miR-1247-5p	3.06	0.044
miR-489-3p	3.28	0.006
miR-181c-5p	3.75	0.011
miR-3614-5p	3.86	0.026
miR-935	4.05	0.002
Let-7e-3p	4.16	0.012
miR-181b-5p	4.36	0.001
miR-27b-5p	4.49	0.029
miR-181a-5p	5.91	0.001
miR-615-3p	7.33	0.016
miR-181a-3p	19.6	0.000

Differential expression of miRNAs in MCF-7-AR6 presented as fold change. Positive fold-change denotes upregulation while negative fold change denotes downregulation. ANOVA, analysis of variance.
